# Missense variant contribution to *USP9X*-female syndrome

**DOI:** 10.1038/s41525-020-00162-9

**Published:** 2020-12-09

**Authors:** Lachlan A. Jolly, Euan Parnell, Alison E. Gardner, Mark A. Corbett, Luis A. Pérez-Jurado, Marie Shaw, Gaetan Lesca, Catherine Keegan, Michael C. Schneider, Emily Griffin, Felicitas Maier, Courtney Kiss, Andrea Guerin, Kathleen Crosby, Kenneth Rosenbaum, Pranoot Tanpaiboon, Sandra Whalen, Boris Keren, Julie McCarrier, Donald Basel, Simon Sadedin, Susan M. White, Martin B. Delatycki, Tjitske Kleefstra, Sébastien Küry, Alfredo Brusco, Elena Sukarova-Angelovska, Slavica Trajkova, Sehoun Yoon, Stephen A. Wood, Michael Piper, Peter Penzes, Jozef Gecz

**Affiliations:** 1 University of Adelaide and Robinson Research Institute, Adelaide, SA 5005 Australia; 2 Department of Physiology, Northwestern University Feinberg School of Medicine, Chicago, Il USA; 3 Women’s and Children’s Hospital, Adelaide, SA 5006 Australia; 4 South Australian Health and Medical Research Institute, Adelaide, SA 5000 Australia; 5 Hospital del Mar Research Institute (IMIM), Network Research Centre for Rare Diseases (CIBERER) and Universitat Pompeu Fabra, Barcelona, 08003 Spain; 6 Institut Neuromyogène, métabolisme énergétique et développement durable, CNRS UMR 5310, INSERM U1217, Université de Lyon, Université Claude Bernard Lyon 1, Lyon, France; 7 Service de Génétique, Hospices Civils de Lyon, Lyon, France; 8 Division of Genetics, Department of Pediatrics, University of Michigan, Ann Arbor, MI USA; 9 Section of Neurology, Department of Pediatrics, St. Christopher’s Hospital for Children, Drexel University College of Medicine, Philadelphia, PA USA; 10 Division of Clinical Genetics, Department of Pediatrics, Columbia University Irving Medical Center, New York, NY USA; 11 Dr. von Hauner Children’s Hospital, LMU - Ludwig-Maximilians-Universität Munich, University of Munich Medical Center, Munich, Germany; 12 Kingston Health Sciences Centre, Kingston, ON K7L 2V7 Canada; 13 Division of Medical Genetics, Department of Pediatrics, Kingston General Hospital, Kingston, ON Canada; 14 Division of Genetics and Metabolism, Children’s National Hospital, Washington, DC USA; 15 Unité Fonctionnelle de génétique clinique, Hôpital Armand Trousseau, Assistance publique-Hôpitaux de Paris, Centre de Référence Maladies Rares des anomalies du développement et syndromes malformatifs, Paris, France; 16 Hôpital de la Pitié-Salpêtrière, Département de Génétique, Paris, France; 17 Division of Genetics, Department of Pediatrics, Medical College of Wisconsin, Milwaukee, WI USA; 18 Victorian Clinical Genetics Service, Melbourne, VIC Australia; 19 Department of Paediatrics, University of Melbourne, Melbourne, VIC Australia; 20 Murdoch Children’s Research Institute, Melbourne, VIC Australia; 21 Department of Human Genetics, Donders Institute for Brain, Cognition and Behaviour, Radboud University Medical Center, Nijmegen, 6500 HB the Netherlands; 22 Service de Génétique Médicale, CHU Nantes, 44093 Nantes, France; 23 l’Institut du Thorax, INSERM, CNRS, UNIV Nantes, 44007 Nantes, France; 24 Department of Medical Sciences, University of Turin, Torino, Italy; 25 Medical Genetics Unit, Città della Salute e della Scienza University Hospital, Torino, Italy; 26 Department of Endocronology and Genetics, University Clinic for Children’s Diseases, Medical Faculty, University Sv. Kiril i Metodij, Skopje, Republic of Macedonia; 27 Griffith Institute for Drug Discovery, Griffith University, Brisbane, QLD Australia; 28 School of Biomedical Sciences, University of Queensland, Brisbane, QLD 4072 Australia; 29 Queensland Brain Institute, The University of Queensland, Brisbane, QLD 4072 Australia

**Keywords:** Neurodevelopmental disorders, Neurodevelopmental disorders, Development, Genetics research

## Abstract

*USP9X* is an X-chromosome gene that escapes X-inactivation. Loss or compromised function of *USP9X* leads to neurodevelopmental disorders in males and females. While males are impacted primarily by hemizygous partial loss-of-function missense variants, in females de novo heterozygous complete loss-of-function mutations predominate, and give rise to the clinically recognisable *USP9X*-female syndrome. Here we provide evidence of the contribution of *USP9X* missense and small in-frame deletion variants in *USP9X*-female syndrome also. We scrutinise the pathogenicity of eleven such variants, ten of which were novel. Combined application of variant prediction algorithms, protein structure modelling, and assessment under clinically relevant guidelines universally support their pathogenicity. The core phenotype of this cohort overlapped with previous descriptions of *USP9X*-female syndrome, but exposed heightened variability. Aggregate phenotypic information of 35 currently known females with predicted pathogenic variation in *USP9X* reaffirms the clinically recognisable *USP9X*-female syndrome, and highlights major differences when compared to *USP9X*-male associated neurodevelopmental disorders.

## Introduction

The deubiquitylating enzyme encoded by *USP9X* is best known for its roles in brain development and cancer^1^. It is ranked among the top 5% of evolutionary constrained genes and is highly intolerant to variation (pLI = 1.0; z-score = 6.35)^1–4^. It is essential for embryonic viability^5^. USP9X functions to reverse the effects of protein ubiquitylation, a frequent post-translational modification that often culminates in protein degradation via the proteasome^6^. USP9X thus protects many of its substrates from degradation, thereby increasing their abundance and hence function^1^. Many USP9X substrates are encoded by genes involved in brain development and neurodevelopmental disorders (NDDs)^1^. Furthermore, rare *USP9X* mutations have been identified to directly cause NDDs^7–10^.

*USP9X* is located on the X-chromosome. The inheritance patterns and clinical presentations of the X-linked disorders often differ between males and females. X-linked disorders predominantly affect hemizygous males while female carriers are generally unaffected. This was the case for the historical family MRX99, with multiple affected males, in which a C-terminal protein truncating variant in *USP9X* was maternally transmitted across three generations^8^. An additional 15 likely pathogenic missense variants have since been reported in affected males, with further 26 of uncertain significance (VUS)^7,8^. Many of these 42 variants were inherited through unaffected mothers, while others arose de novo. We showed that these male *USP9X* missense variants cause partial, rather than complete loss of USP9X function^7,8,11^. In particular, these mutations disable brain-specific USP9X functions, while leaving other functions, such as those essential for embryonic viability, intact.

Sparing of heterozygous females in X-linked disorders may involve protective X-inactivation. *USP9X* is, however, an atypical X-chromosome gene which escapes X-inactivation, and thus the likely mechanisms is also related to its gene expression and as such dosage^10^. Nonetheless, the *USP9X* mutations found in the affected females are predominantly complete loss of function (LOF) alleles. In total, 20 females have been reported with a syndromic NDD, also known as MRX99F, caused by de novo heterozygous *USP9X* null mutations including whole and partial gene deletions, nonsense and early frameshift mutations^10,12–14^. Studies using patient derived cells established that these LOF mutations caused reduction of *USP9X* mRNA and protein abundance in affected females, suggesting haploinsufficiency as a likely disease mechanism. Such LOF alleles are likely to never be observed in a male as complete loss of *USP9X*, as would be the case of a hemizygous male, is known to result in early embryonic lethality in at least mouse^5^.

USP9X is significantly depleted of missense variants, which would indicate that at least some of these variants are likely highly deleterious to USP9X function, e.g. when located in critical domains of the protein. Indeed, a single missense mutation altering a residue in the catalytic domain of USP9X has been identified in a female individual with strong clinical resemblance to others with *USP9X*-female syndrome^10^. In this report, we identify and study an additional 10 novel female variants, 8 missense and 2 in-frame single amino acid deletions. In depth comparative *in silico* predictions and structural modelling provided support of pathogenicity. We also assess and compare the clinical presentations of this cohort against all reported individuals with *USP9X*-female syndrome. We define an expanded and overlapped phenotypic spectrum of these female cohorts, which collectively revealed similarities and differences in the phenotypic features observed in male versus female *USP9X* associated NDDs. Collectively, our data expands the mutational mechanisms and phenotypic outcomes relating to *USP9X*-female syndrome.

## Results

### Identification of novel USP9X variants in affected females

Following our earlier reports of *USP9X* variants in male and female cases with NDDs^8,9^, we have collected, through international clinical, diagnostic and research centres, several additional female ascertained *USP9X* variants of unknown clinical significance. As part of this study we have further selected and scrutinised eight missense variants and two in-frame single amino acid deletions. These variants were predominantly identified using trio based exome sequencing (Table 1; Supplementary Data). In addition to these 10 novel variants we also analysed one previously reported missense variant (Female 8; ref. ^10^).

**Table 1:** Details of *USP9X* missense and single amino acid deletion variants associated with *USP9X*-female syndrome.

Case ID	cDNA	Protein	Catalytic domain	Diagnostic test	Inheritance	gnomaD	ACMG	Polyphen_2	CADD
Female 21	c.671 T > C	p.Leu224Pro	No	DES	De novo	0	LP	D	29.9
Female 22	c.1073 T > A	p.Val358Asp	No	WES–Trio	De novo	0	LP	D	26.4
Female 23	c.1303 T > C	p.Trp435Arg	No	WES–Trio	De novo	0	LP	P	27.1
Female 24	c.3664 G > C	p.Ala1222Pro	No	WES–Trio	De novo	0	LP	D	29.5
Female 25	c.3986 G > A	p.Arg1329His	No	WES–Trio	De novo	0	LP	D	32
Female 34	c.4147_4149delCTT	p.Leu1383del	No	WES–Trio	De novo	0	LP	n/a	21.3
Female 26	c.5053 G > A	p.Asp1685Asn	Yes	WES–Trio	Maternal^a^	0	LP	D	29.7
Female 27	c.5053 G > A	p.Asp1685Asn	Yes	WES–Trio	De novo	0	LP	D	29.7
Female 8	c.5078 T > G	p.Leu1693Trp	Yes	WES–Trio	De novo	0	LP	D	28.7
Female 33	c.5290 G > A	p.Glu1764Lys	Yes	WES–Trio	Maternal^b^	0	LP	D	32
Female 28	c.5405 A > G	p.Tyr1802Cys	Yes	WES–Trio	De novo	0	LP	D	29.9
Female 29	c.5642_5644delATT	p.Tyr1881del	Yes	WES–Trio	De novo	0	LP	n/a	22.4

cDNA coordinates are given in reference to NM_001039590.2 and protein coordinates in reference to NP_001034679.2.

*DES* disease exome sequencing, *WES–Trio* whole-exome sequencing in Trio, *gnomAD* Genome Aggregation Database V2.1.1, *ACMG* American College of Medical Genetics classification^15^ where LP stands for likely pathogenic; Polyphen_2 represents prediction based on HDIV scores where D: damaging and P: possibly damaging; Combined Annotation Dependent Depletion (CADD) scores are given where CADD >20 equates to the top 1% of deleterious variants^17^. *n/a* not available.

^a^Maternal germline mosaic.

^b^Non mosaic (at least in blood).

**Fig. 1:**
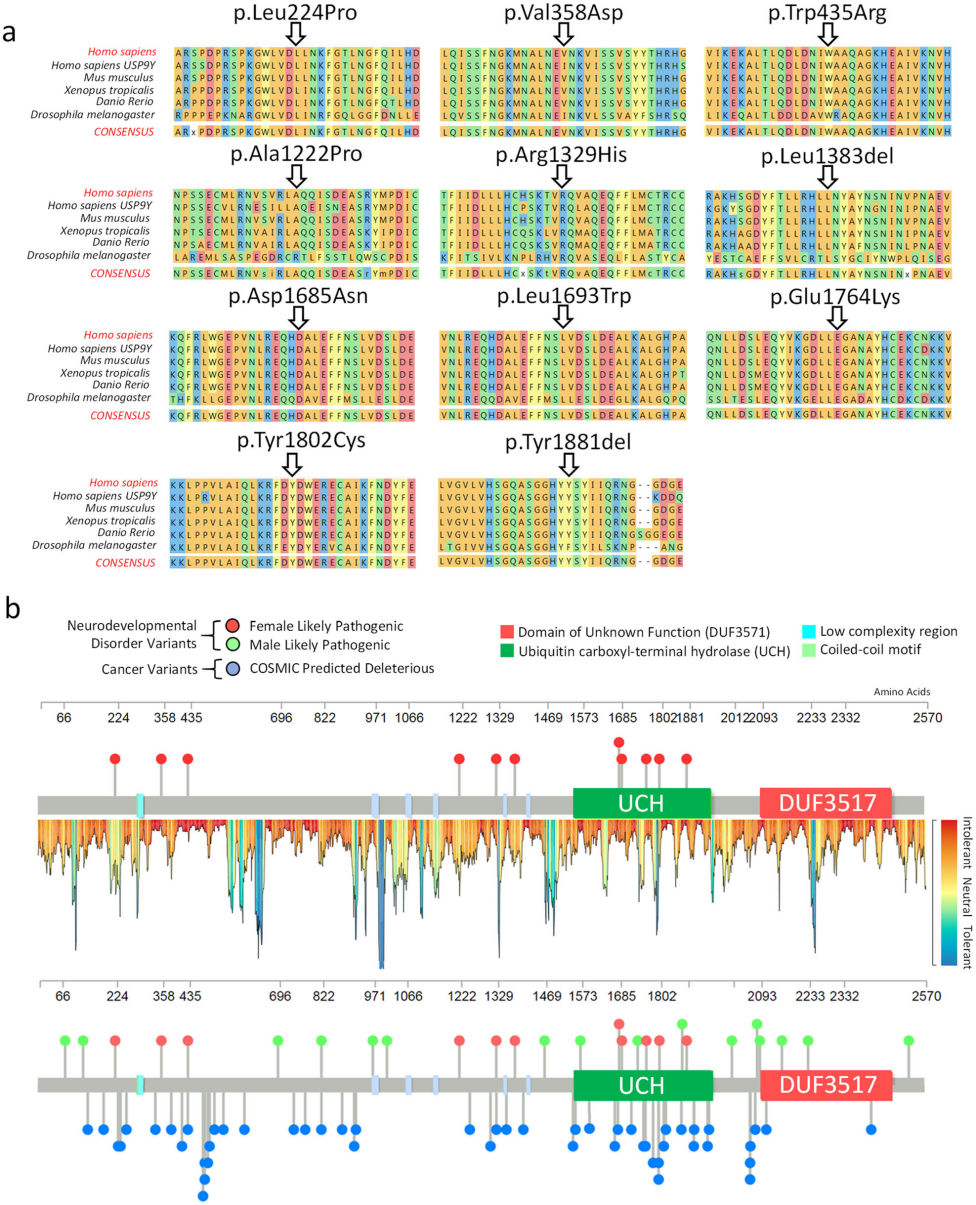
Conservation and protein location of likely pathogenic USP9X-female variants. **a** Cross species protein alignment of *USP9X* showing conservation of altered amino acid residues. **b** Location of female likely pathogenic variants on the USP9X protein structure. USP9X variation tolerance landscape is provided (see Materials and Methods). Locations of male likely pathogenic variants and predicted deleterious missense somatic cancer variants (extracted from COSMIC database with CADD score ≥30) are shown for comparison.

One of the variants (p.Asp1685Asn) was found in two unrelated individuals (Females 26 and 27). In Female 26, this variant was inherited from a mosaic mother (de novo in mother). A second inherited variant in Female 33 (p.Glu1764Lys) was also passed on from the mother, but intriguingly with no evidence of mosaicism, at least in blood (Table 1 and Supplementary Data). This variant was not found in the maternal grandmother, while the maternal grandfather (with no signs of disability) could not be tested. The other nine of eleven variants all arose de novo (Supplementary Data). Applying the guidelines of the American College of Medical Genetics Guidelines (ACMG; ref. ^15^) we classified all of these 11 variants as likely pathogenic (Table 1 and Supplementary Data). All were also predicted to be deleterious using Polyphen2^16^ and Combined Annotation Dependent Depletion (CADD; ref. ^17^) algorithms (Table 1 and Supplementary Data). All 11 variants alter highly conserved amino acids, all but one invariable (Fig. 1a). These variants are novel and not present in Genome Aggregation Database (gnomAD V2.1.1;^18^) consisting of >140,000 exomes or genomes (Table 1). In general, the variants impacted regions of the USP9X protein, which were predicted to be intolerant to variation (Fig. 1b; ref. ^19^). Five of the variants were located in the ubiquitin C hydrolase (UCH) catalytic domain, while the others were found distributed in the N-terminal extension of the protein of largely unknown structure and function (Table 1 and Fig. 1b). To see if these variants clustered in potential *USP9X* ‘mutation hotspots’, we compared their location with those of likely pathogenic variants associated with male NDDs (*n* = 16; ref. ^7^). Furthermore, as *USP9X* loss-of-function mutations are also enriched in somatic cancers^1^, we included variants extracted from the Catalogue of Somatic Mutations in Cancer (COSMIC) database that are predicted to be deleterious (CADD score >30; *n* = 49). While this analysis did not reveal any striking variant ‘hotspots’, we noted that the catalytic domain was potentially enriched with female variants, and enriched with cancer variants, compared to its flanking regions (*p* = 0.08 and *p* = 0.00017 respectively via two-proportion z-test; Fig. 1b).

Altogether we identify 10 novel female *USP9X* missense and single amino acid deletion variants associated with NDDs, which alter generally invariable amino acids and are located in protein regions required for catalytic activity and/or intolerant to variation.

### Variant prediction algorithms support pathogenicity of *USP9X*-female missense variants

**Fig. 2:**
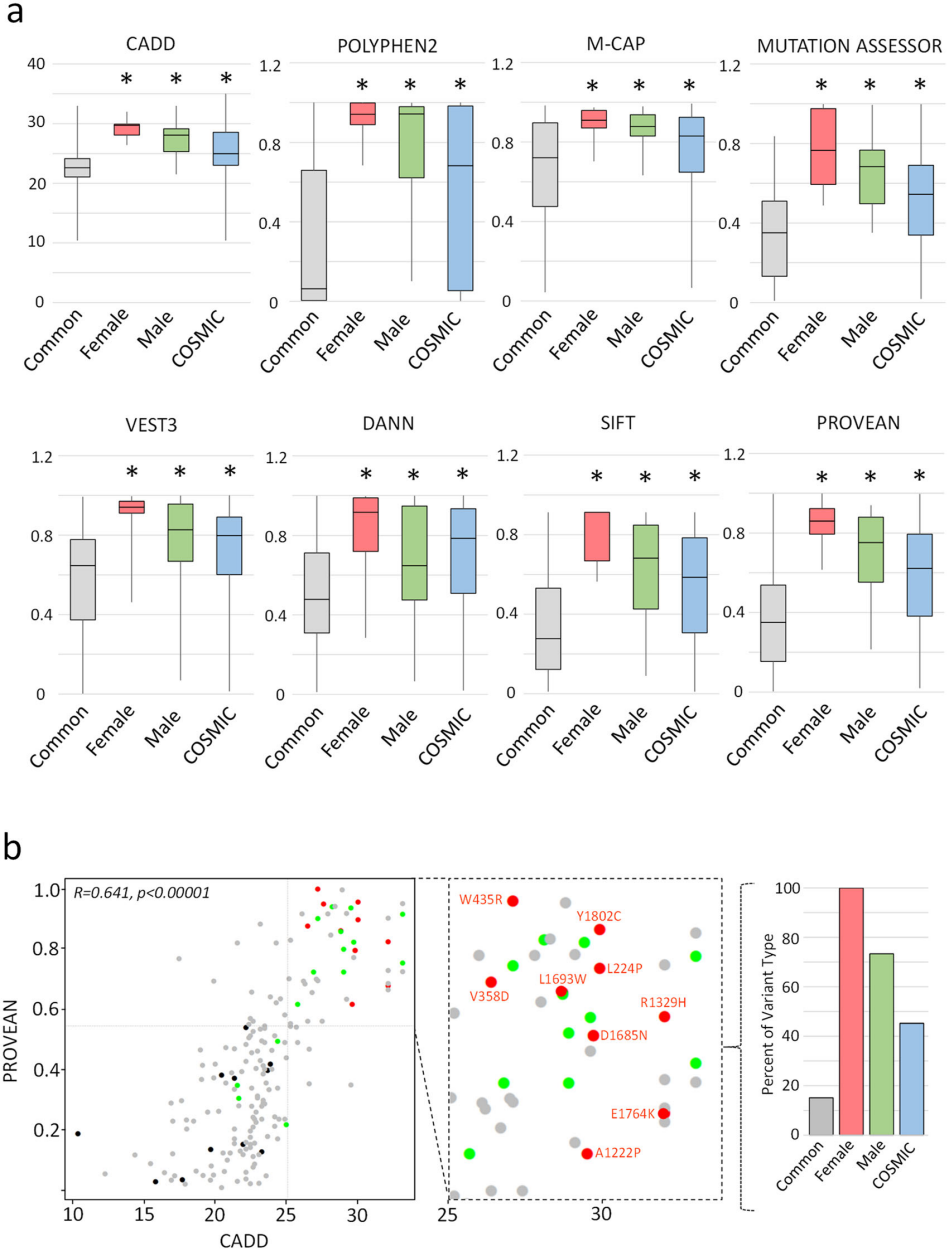
*USP9X*-female missense variants display in silico signatures of pathogenicity. **a** Aggregate comparison of common variants extracted from gnomAD (allele frequency >1:100000), against females missense variants, likely pathogenic male missense variants, and variants found in cancer (extracted from COSMIC database) using a suite of in silico prediction tools. Box-whisker plots are defined as follows: centre line, median; box limits, upper and lower quartiles; whiskers, min and max values. *significantly different from common variants *p* < 0.05 by two-tailed equal variance Student’s *t*-test. **b** Comparison of CADD and PROVEAN scores reveal clustering of variants all female missense variants in the upper-right quadrant consistent with pathogenicity (CADD > 25, PROVEAN > 0.565). Scores of common variants are significantly correlated (Pearson’s correlation given). Colour scheme as in **a**. Inset identifies each variant in the ‘pathogenic quadrant’. Graphs show percent of each type of variant, and the overall composition of variant types within the pathogenic quadrant.

To extend our investigations into the functional (or not) effect of these variants, we employed an array of in silico missense variant pathogenicity predictive tools. We compared the outcomes of these predictive algorithms between the female likely pathogenic missense variants and that of common (i.e. assuming to be benign) *USP9X* missense variants. We defined a common variant as one with an allele frequency >1:100,000 in the gnomAD V2.1.1 database (*n* = 159)^18^. The gnomAD derived variants are from individuals devoid of severe paediatric disease, and are found distributed throughout the entire protein (including catalytic domain and regions predicted intolerant to variation). They provide the best, currently available control dataset to further interrogate the pathogenicity of *USP9X* variation (Supplementary Fig. 1). We submitted all missense variants, including common variants, female and male NDD variants, and all COSMIC variants (*n* = 358; Supplementary Fig. 1) to the suite of variant prediction tools within the ANNOVAR ensemble^20^. We previously established several tools with the best predictive power for assessing *USP9X* missense variation^7^. Using these same tools, we compared the combined predictive scores of the *USP9X* common variants with the female, male and cancer variants. For each tool used (CADD, Ployphen2, M-CAP, Mutation Assessor, VEST3, DANN, SIFT and PROVEAN), female missense variants scored significantly higher than common variants, thus supporting pathogenicity (Fig. 2a). The scores of female variants also trended higher than male variants, and that of cancer, suggesting that on average they were more deleterious. We have also reported beforehand that a combined score of CADD and PROVEAN provides a highly stringent predictive rubric for *USP9X* missense variation^7^. Applying this approach, we found 100% of these nine female missense variants tested were scored as pathogenic (CADD > 25 and PROVEAN > 0.5665) compared to 75% of male likely pathogenic variants, 45% of cancer variants and only 15% of common variants (Fig. 2b). Thus, several in silico predictive approaches provided congruent support of the pathogenicity of female *USP9X* likely pathogenic missense variants.

### Protein structure modelling of the USP9X catalytic domain variants

**Fig. 3:**
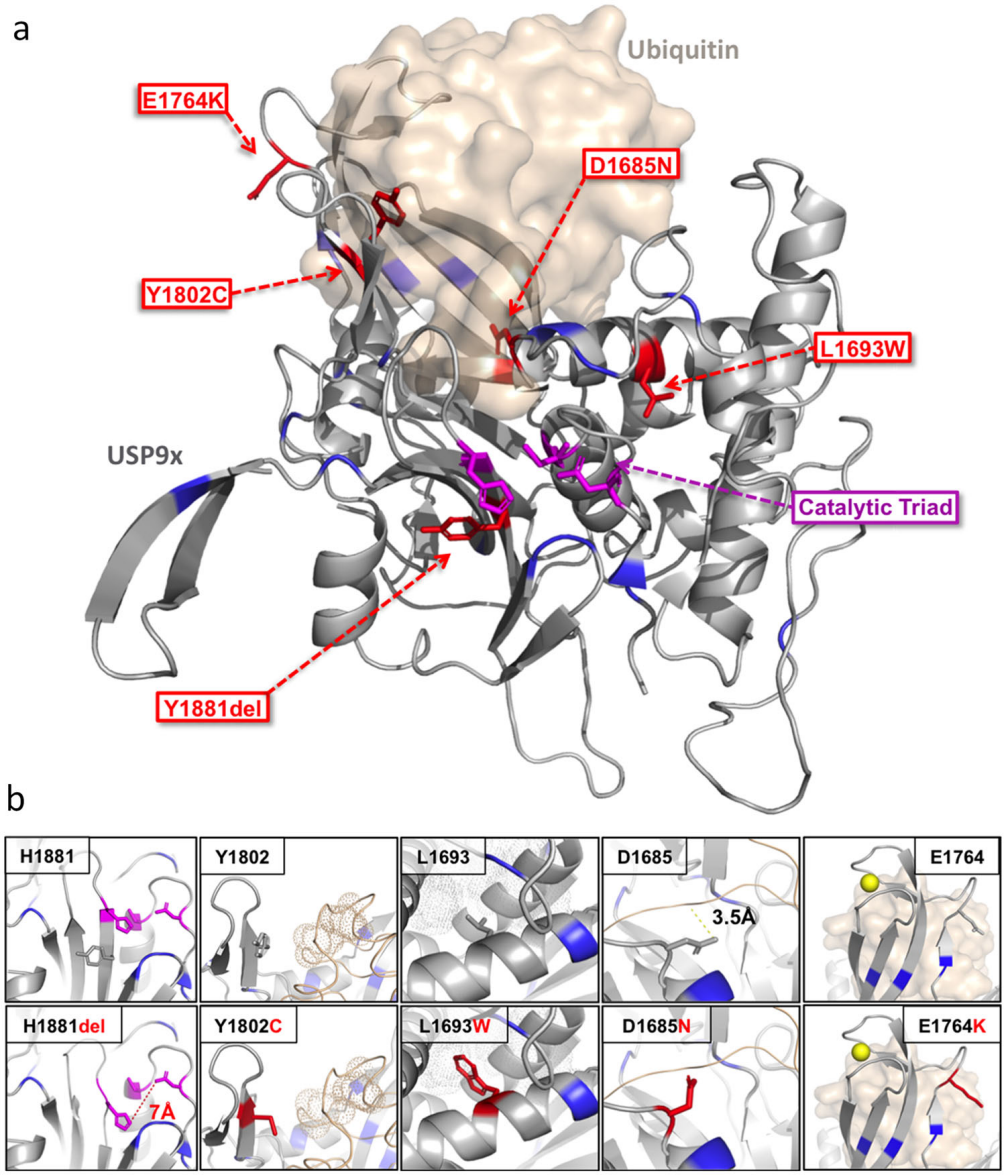
Structural modelling of *USP9X*-female variants located in the catalytic domain. **a** Homology model of USP9X (grey) with catalytic site (magenta), likely pathogenic female variants (red) and location of predicted deleterious cancer variants (blue; extracted from COSMIC database with CADD score >30). Interaction with ubiquitin is shown. Likely pathogenic variants are positioned in regions of well‐ordered secondary structure or flexible regions involved in zinc-binding. **b** Insets indicate local structural effects of indicated likely pathogenic female USP9X variants. All native amino acid side chains are represented as grey sticks. Variant amino side chains are indicated by red sticks. Side chains of the amino acids forming the core catalytic site are indicated by magenta sticks. Location of cancer variants is highlighted in blue. Zinc ion is represented with a yellow sphere. Hydrophobic van der vaals radii are indicated by dots and charge–charge interactions are shown by dotted lines.

We employed our recently resolved USP9X UCH catalytic domain structural model^7,11^ to interrogate the molecular mechanisms underpinning pathogenicity of the five female missense and single amino acid deletion variants found within (Table 1 and Fig. 3). Notably, the predicted effects of missense variation using this structural model thus far have been validated using in vitro recombinant protein binding and deubiquitinating assays^11^. The p.Tyr1881 residue deleted in Female 29 contributes to a beta-sheet critical for the positioning of the UCH catalytic triad. The p.Tyr1881 deletion is predicted to alter the position of the catalytic residue p.His1879 and likely to have significant effects on catalytic activity. Predicted deleterious cancer variants (i.e. CADD > 30) in close proximity (p.Ser1872Asn, p.Ala1875Val, p.Ser1876Gly, p.Val1870Ile) likely act via similar mechanisms (Fig. 3). The p.Tyr1802 residue altered in Female 28 contributes to the hydrophobic surface involved in ubiquitin binding via interaction with the p.Ile36 residue of ubiquitin. The p.Tyr1802Ser substitution introduces a polar amino acid predicted to disrupt the hydrophobicity and ubiquitin binding. Predicted deleterious cancer variants (p.Lys1798Thr, p.Arg1799Gln, p.Arg1799Leu) are proposed to have similar effect (Fig. 3). The p.Asp1685Asn substitution in Female 27 also results in an amino acid charge reversal, and is predicted to alter the intramolecular charge–charge interaction with p.Gln1796, and as such constrict the ubiquitin binding channel and sterically clash with the backbone amine of the p.Leu73 residue of ubiquitin. This mechanism is similar to that predicted for proximal deleterious cancer variants (p.Glu1688Lys, p.Glu1688Ala; Fig. 3). The p.Leu1693Trp variant in Female 8 introduces a highly bulky tryptophan predicted to disrupt the local hydrophobic core provided by p.Val1643, p.Leu1647, p.Phe1689 and p.Phe1671. The importance of maintaining this fold is highlighted by the presence of predicted deleterious cancer variants acting via similar mechanism (p.Ser1692Leu and p.Val1694Met; Fig. 3). The p.Glu1764Lys variant in Female 33 lies within the zinc finger motif of the catalytic domain, which forms multiple contacts with ubiquitin and is integral to the catalytic activity of several related deubiquitinating enzymes^21,22^. Indeed, structure based mutations of USP9X which disrupt zinc-binding alter its activity towards specific types of ubiquitin chain linkages^23^, while the presence of multiple deleterious cancer variants (p.Asp1761Tyr, p.Asp1720Asn and p.Arg1783Cys) in the zinc finger motif provide additional support of its importance. Thus while p.Glu1764Lys is surface exposed and not likely to be involved in stabilizing intramolecular interactions, its close proximity to the zinc-binding site suggests it may alter the positioning or zinc-binding properties of this motif, suggesting profound effect on USP9X ubiquitin chain specificity and activity^23^. Thus structural modelling of the all likely pathogenic USP9X-female variants located in the catalytic domain provides rationale for disrupted catalytic activity and/or ubiquitin binding, and is supported by analogous mechanisms of several proximal predicted deleterious variants arising in cancer.

### Variable phenotype of females with USP9X missense variants

**Fig. 4:**
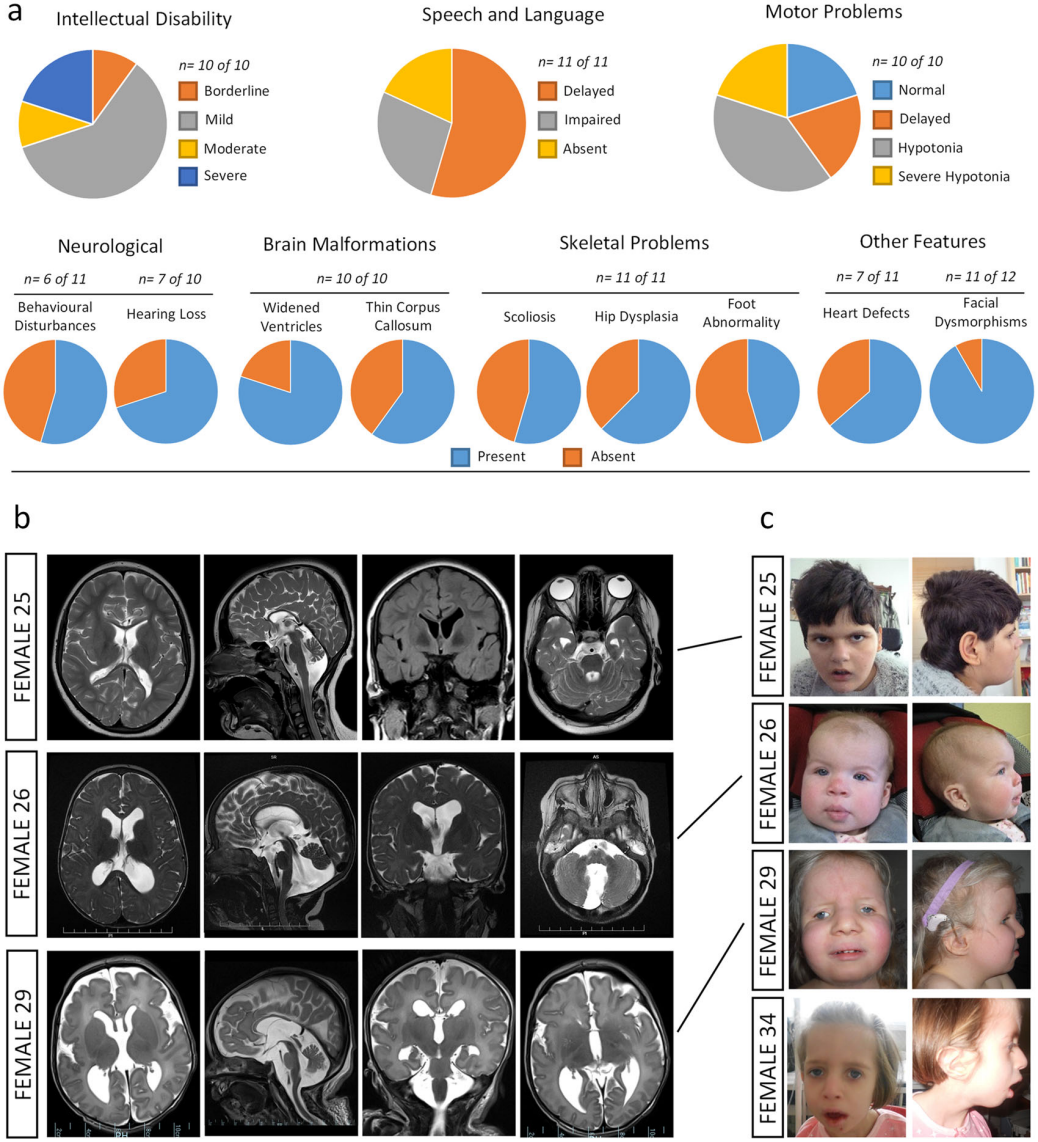
Phenotypic features of females with *USP9X* missense and single amino acid deletion variants. **a** Constellation and frequency of major clinical features. **b** Magnetic resonance imaging (MRI) of brains of affected individuals. Note prominent extra-axial spaces in all, hypoplastic corpus callosum in Females 25 and 29, optic nerve atrophy and in Female 25, Dandy Walker malformation and Blake’s pouch cyst in Female 26, and ventriculomegaly of the 3rd and 4th ventricles in Female 29. **c** Images of affected females showing facial dysmorphisms, with common features including deep-set eyes, telecanthus, blepharophimosis, a broad nasal tip with wide alae and short collumnella, and low set and dysplastic ears. Written consent was obtained for the publication of photographs.

All females in our missense and single amino acid deletion cohort (*n* = 12) were ascertained primarily on the basis of psychomotor developmental delay (Fig. 4a and Supplementary Data). Intellectual disability (ID) was present in all individuals where assessed, but was variable, ranging from borderline to severe. All individuals displayed problems with speech and language, the severity of which was also across a wide spectrum, ranging from somewhat innocuous delay through to complete absence. There was also variable effects on the development of motor function, ranging from unreported to severe disability, which in two individuals required standing supports or wheel chairs. Motor disability was most frequently related to hypotonia (Fig. 4a). Hearing loss was a prominent feature, and individuals displayed a number of different behavioural disturbances including autism, anxiety and aggression (Fig. 4a and Supplementary Data). All individuals presented with brain malformations, most frequently agenesis of the corpus callosum and ventriculomegaly (Fig. 4a, b and Supplementary Data). Other congenital abnormalities were also observed, involving skeletal defects affecting the spine, feet and hips, and heart defects, the latter of which resulted in neonatal lethality in one case (Female 23; Supplementary Data). In addition, abnormalities affecting skin, gastroenterological, urogenital, metabolic and endocrine systems were observed at lower frequencies (Supplementary Data). Facial dysmorphisms were prevalent in almost all individuals, and was in close alignment with previously reported individuals with *USP9X*-female syndrome, with common features including deep-set eyes, telecanthus, blepharophimosis, broad nasal tip with wide alae and short collumnella, low set and dysplastic ears, small mouth and micrognathia (Fig. 4a, c, Supplementary Fig. 2 and ref. ^10^). These data reveal a variable phenotype associated with missense and single amino acid deletion *USP9X* variants in females, with major features of developmental delay, ID, speech and motor delay, prevalent brain malformations and other congenital aspects particularly affecting the development of the craniofacial structures spine and heart.

### Affected females share key clinical presentations, which differ in USP9X males

**Fig. 5:**
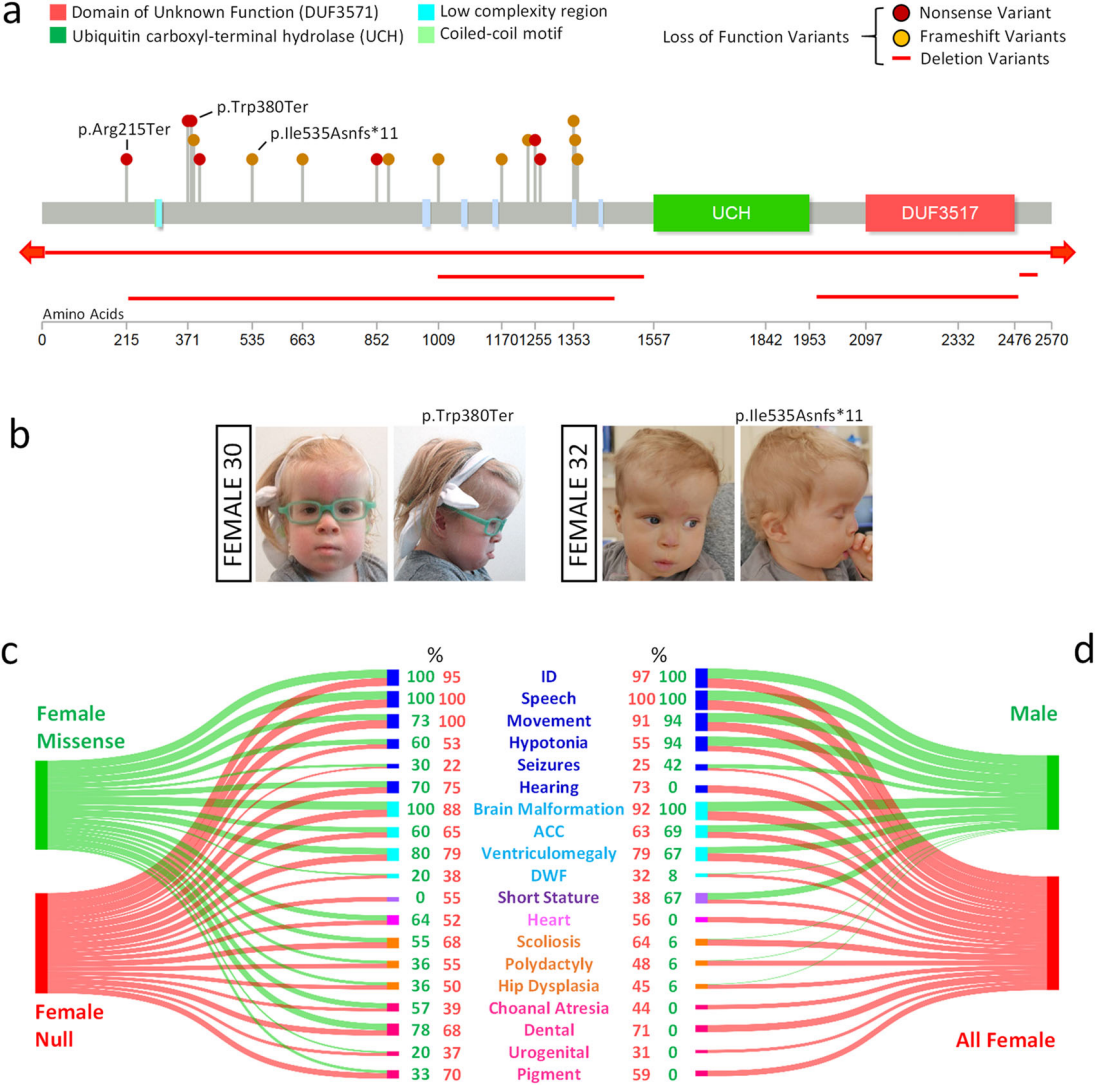
Comparison of phenotypic features of females with different *USP9X* variant types and with males. **a** Location of bona fide loss-of-function variants in individuals with *USP9X*-female syndrome. Three novel variants described in this study are annotated. **b** Images showing facial dysmorphisms of females affected by novel nonsense and frameshift *USP9X* variants. Note resemblance to individuals in Fig. 4c. **c** Comparison of clinical features of females with missense and single amino acid deletions (*n* = 12) variants with females harbouring null alleles including all previously published cases and three novel cases identified in this study (*n* = 23). Sankey plot highlights overlap across all features except short stature. **d** Comparison of clinical features of a combined female cohort (*n* = 35) with phenotypes of all published males with likely pathogenic variants (*n* = 16). Sankey plot highlights overlap across neurological features but not in other major female associated congenial phenotypes. Thickness of each stream is proportional to the percentage of cases with each feature, which is also provided numerically at the terminal nodes.

We also sought to establish if the clinical presentations of our female cohort with missense and single amino acid deletion variants overlapped with those of previously reported individuals with *USP9X*-female syndrome caused by bona fide complete loss of function alleles. For this purpose we combined the phenotypic information from the 20 reported individuals with heterozygous gene deletion, nonsense and frameshift *USP9X* alleles (Supplementary Fig. 3; refs. ^10,12–14^). We herein further expand this cohort by reporting an additional three novel cases (Females 30–32; Fig. 5a, b, Supplementary Fig. 2). These individuals presented with phenotypes that also highlight the range of severities associated with *USP9X* variation (Supplementary Data). Female 30 had a *de novo* nonsense variant (p.Trp380Ter) and presented with severe ID, absent speech and severe motor disability. In comparison, Female 32 with a *de novo* frameshift variant (p.Ile535Asnfs*11) had only slight delays in speech, language and motor skills, and is now largely meeting developmental milestones. Furthermore a maternally inherited nonsense variant (p.Arg215Ter) was found following the genetic autopsy of Female 31, a terminated foetus with brain malformation, heart and skeletal defects (Supplementary Data). In this instance, while the mother was assessed as having a history of scoliosis and partial hearing impairment, she was otherwise normal and with no dysmorphic features or additional congenital anomalies. These cases further expand the clinical spectrum associated with bona fide heterozygous loss of function *USP9X* variants.

We then combined phenotypical data from all such individuals (*n* = 23; Supplementary Fig. 3) and compared the frequency of major clinical features to that observed in the female individuals harbouring missense and single amino acid deletion variants (*n* = 12 cases; Supplementary Data). We found strong concordance in the prevalence of combined neurological and other congenital features between these cohorts (Fig. 5c). These data strongly support the pathogenicity of missense and single amino acid deletion variants in females.

Given this strong overlap, we combined data from both these two female cohorts (*n* = 23 + 12 = 35 individuals) to best define the clinical presentations in females with deleterious *USP9X* variants (Fig. 5c). Subsequently, we also used this data to compare females with males (*n* = 16^7^) with pathogenic or likely pathogenic *USP9X* variants (Fig. 5d). While the major neurological findings were similar between males and females, males were unlikely to have congenital presentations including skeletal and heart defects, among others (Fig. 5d). These data also support the deleterious effect of the *UPS9X* missense and single amino acid deletion variants, refine *USP9X*-female syndrome phenotype from an aggregate of 35 individuals, and point to major differences between males and females with *USP9X* variants.

## Discussion

Here we expand the genotypic and phenotypic spectrum of *USP9X*-female syndrome. The current state of knowledge suggests that de novo heterozygous complete LOF *USP9X* variants cause a defined syndrome in heterozygous females^10^. Twenty-three such females have been now been reported, including the three novel cases herein. These female LOF mutations include whole or partial gene deletions, early nonsense and frameshift mutations^10,12–14^. Males with such LOF mutations are unlikely to survive early stages post fertilisation^5^. Here we present new evidence that missense and single amino acid deletion variants can also cause a similar female phenotype. Prior to this study, only a single affected female missense variant located in the USP9X catalytic domain had been reported^10^. We now add 10 additional variants classified as likely pathogenic under the ACMG guidelines, shedding new light into the genetic origin of *USP9X* disease as well as its clinical presentations.

Eight of these 10 novel variants occurred *de novo* in these females, which by a traditional view could also have been considered an ‘incidental finding’ of an *USP9X* unaffected carrier female. One individual had an inherited variant from their mother who were subsequently found to be mosaic, a mode which has been previously reported^10^. The remaining case of Female 33, with a variant inherited from her unaffected mother, who is unlikely a somatic mosaic, suggests possible modifier(s) of female penetrance, perhaps similar to e.g. the X-chromosome linked *PCDH19* clustering epilepsy^24^. While it is also plausible to question this specific *USP9X* variant’s pathogenicity, the variant is located within the catalytic domain, it is predicted to be pathogenic universally by several algorithms, and the affected girl bears clear clinical resemblance to *USP9X*-female syndrome. That includes cardinal clinical presentations such as skin pigmentation, hip dysplasia, heart defects, choanal atresia and hearing loss in addition to other hallmark neurological and craniofacial features. We also identified another similar transmission, in a case of a maternally inherited nonsense variant (Female 31). In this instance the LOF effect of this variant (p.Arg215*) is highly likely. The carrier mother of this child had a history of scoliosis, but was otherwise unaffected. These two cases of variable penetrance of *USP9X* LOF variants in heterozygous females suggest the existence of a disease modifier. One possible candidate is skewing of X-inactivation, but *USP9X* is known to escape X-inactivation. That said, the degree to which a gene escapes from X-inactivation is known to be variable, and can be tissue specific^25–28^. Complete skewing of X-inactivation may also suggest the existence of another genetic abnormality on one of the X-chromosomes of the affected female, which can be contributing to the phenotype independently of *USP9X*, e.g. as has been observed in an affected female carrier of Fragile X^29^. Furthermore, a haploinsufficiency-like mechanism of *USP9X*-female NDD is not supported by e.g. phenotypes observed in Turner Syndrome with XO sex chromosome karyotype, which generally lack neurological manifestations^30^. Possible role for X-inactivation in *USP9X*-female NDDs is suggested by several frequently observed clinical features including mosaic skin pigmentations and asymmetries in brain formation, breast development, limb development and other structures^10,12,13^. Previous studies have however shown that skewing of X-inactivation in DNA obtained from three out of five patient derived fibroblasts showed no correlation with disease severity^10^. However, as it is also the case in e.g. *PCDH19* clustering epilepsy, where the blood or skin-derived X-inactivation is not informative^31^, mouse model evidence suggests that it is at play in at least brain^32^. X-inactivation studies revealed no evidence of skewing in Female 33, whilst studies on the mothers DNA were uninformative across multiple loci (data not shown). We were also unable to conduct further studies on Female 31 and her mother. Other potential modifiers may relate to specific variants, such as interindividual variation in nonsense mediated mRNA decay^33^ or associated transcriptional compensation^34,35^. Thus examples and identification of *USP9X* variants with variable penetrance offer an opportunity to investigate the likely modifiers and their mechanism in a deeper and more systematic manner.

The set of major clinical features of *USP9X*-female syndrome associated with de novo heterozygous gene deletion, nonsense and frameshift mutations involves developmental delay, ID, brain malformations and other congenital abnormalities impacting craniofacial development, and the heart, skeleton, skin and other organs^10^. These clinical presentations were also observed in our cohort of females with missense and single amino acid deletion *USP9X* variants, but which also appeared variable. Variability in female *USP9X* associated NDDs can also be driven by the underlying mutation type and not just existence of modifiers (as discussed above). All previously reported mutations result in a loss of *USP9X* dosage^10^, with likely downstream impact on all USP9X substrates. This uniform molecular mechanism was proposed to underpin the consistency in the phenotypic outcomes shared among different individuals giving rise to the reports of a recognisable *USP9X*-female syndrome. Furthermore, genetic ablation of *Usp9x* from the developing mouse brain (loss of dosage) provides a strong recapitulation of the neurological phenotypes of these affected females, including hypoplastic corpus callosum, ventriculomegaly, and learning and memory problems^7,11,36,37^. The impact of missense mutations is less defined, and may cause differential impact on downstream substrates and as such phenotypic outcome(s). The variants affecting the catalytic domain are most likely similar to complete LOF alleles, whilst those in the N-terminal extensions may disrupt only subsets of USP9X substrates (see below). It is yet to be determined as to whether these missense variants retain residual USP9X function or act as dominant negative alleles.

The affected female missense and single amino acid deletion variants we identified in this study all passed through rigorous in silico testing which further supported pathogenicity. Detailed structural modelling of the five variants impacting the catalytic domain supplied evidence of altered catalytic activity and/or binding to ubiquitin. This was reinforced by the presence of multiple likely deleterious cancer variants (CADD > 30) in close proximity, which were projected to act via overlapping mechanisms. USP9X is a known tumour suppressor^38–40^ and there also exists a significant enrichment of LOF *USP9X* variants in the COSMIC database^1^. It is noteworthy that childhood malignancy has been reported in two female individuals with *USP9X*-female syndrome^10^, and could potentially be involved in the natural course of the condition. The other variants were all located in the N-terminal region of largely undetermined function^1^. Male *USP9X* variants in the N-terminal regions have been shown to disrupt subsets of USP9X substrate interactions, rather than all^7^. These substrates are, however, critical specifically for the function of neurodevelopmental signalling pathways TGFβ, mTOR, Notch and Wnt^7^, all of which have been shown to be deregulated in the developing brains of mice lacking *Usp9x*^11,37,41–43^. It was also striking to see that males shared much of the neurological phenotypical features shared in our female cohorts, but almost none of the other congenital features. At least some of these male variants are inherited from phenotypically normal mothers, and as such likely better tolerated in a heterozygous state. The female missense mutations reported herein the N-terminal region of USP9X are speculated to be more deleterious than their male counterparts, disrupting more critical (but unknown) USP9X functions or overall USP9X protein structural integrity. On aggregate, the outcomes of the in silico predictive tools not only supported the pathogenicity of the female variants, but also showed that they were more deleterious than male variants. This is a preliminary finding and requires larger validation datasets. Highly deleterious variants are unlikely to ever be identified in males due to probable effect on embryonic viability^5,37^. It was notable that the mosaic mother of Female 26 (p.Asp1685Asn) had a history of five miscarriages.

In conclusion, in this study we identified likely pathogenic missense and single amino acid deletion variants, and additional nonsense and frameshift variants in *USP9X* in affected heterozygous females. The phenotype of these females affirmed that of previous reports, but also highlighted its considerable variability. Our study thus reveals the complexities in the clinical definition, and genetic aetiology of an emerging *USP9X*-female syndrome.

## Methods

### Subjects

This study was approved by the Women’s and Children’s Health Network Human Research Ethics Committee, South Australia, Australia (HREC786–07–2020). All subject information was provided following informed guardian consent. The authors affirm that guardians of human research participants provided written informed consent for publication of images in Figs. 4 and 5 and Supplementary Fig. 2.

### Variant analysis

Two-proportion z-test for enrichment of variants in the catalytic domain was conducted using http://www.sthda.com/english/wiki/two-proportions-z-test-in-r where full length USP9X is 2570 amino acids in length and catalytic domain is 396 amino acids in length. Variant predictions were performed using Annovar^20^ accessed via the webserver http://wannovar.wglab.org/. Box-whisker plots are defined as follows: centre line, median; box limits, upper and lower quartiles; whiskers, min and max values. Statistical significance was assessed using two-tailed equal variance Student’s *t*-test with *p* < 0.05. *USP9X* tolerance to variation landscape was established using methods described in ref. ^19^ and outputted using the Metadome Version 1.0.1 webserver at https://stuart.radboudumc.nl/metadome/dashboard.

### Structural modelling

The USP9X catalytic structural homology model was generated and characterized using Maestro Prime module (Schrodinger, LLC, NY, USA) using the crystal structure 5WCH (http://www.rcsb.org/structure/5WCH) with absent flexible loop regions modelled ab initio through energy minimization^7,11,44^. Homology model image generation and mutagenesis were performed using PyMol V1.8.2.0 (Schrodinger).

### Reporting summary

Further information on research design is available in the Nature Research Reporting Summary linked to this article.

## Supplementary information

Supplementary Information

Supplementary Data

Reporting Summary

## Data Availability

Additional data and materials from this study are available from the authors on reasonable request, subject to compliance with our obligations under human research ethics.
