# Supplementary material for: Missense variant contribution to USP9X-female syndrome
Source: NPJ Genom Med. 2020 Dec 9;5:53. doi: 10.1038/s41525-020-00162-9 (PMC7725775; doi:10.1038/s41525-020-00162-9)
Supplement: Supplementary file 1 — Supplementary Information [file 41525_2020_162_MOESM1_ESM.pdf]

# **Missense variant contribution to *USP9X* female syndrome.**

# SUPPLEMENTARY FIGURES

This file contains 3 Figures

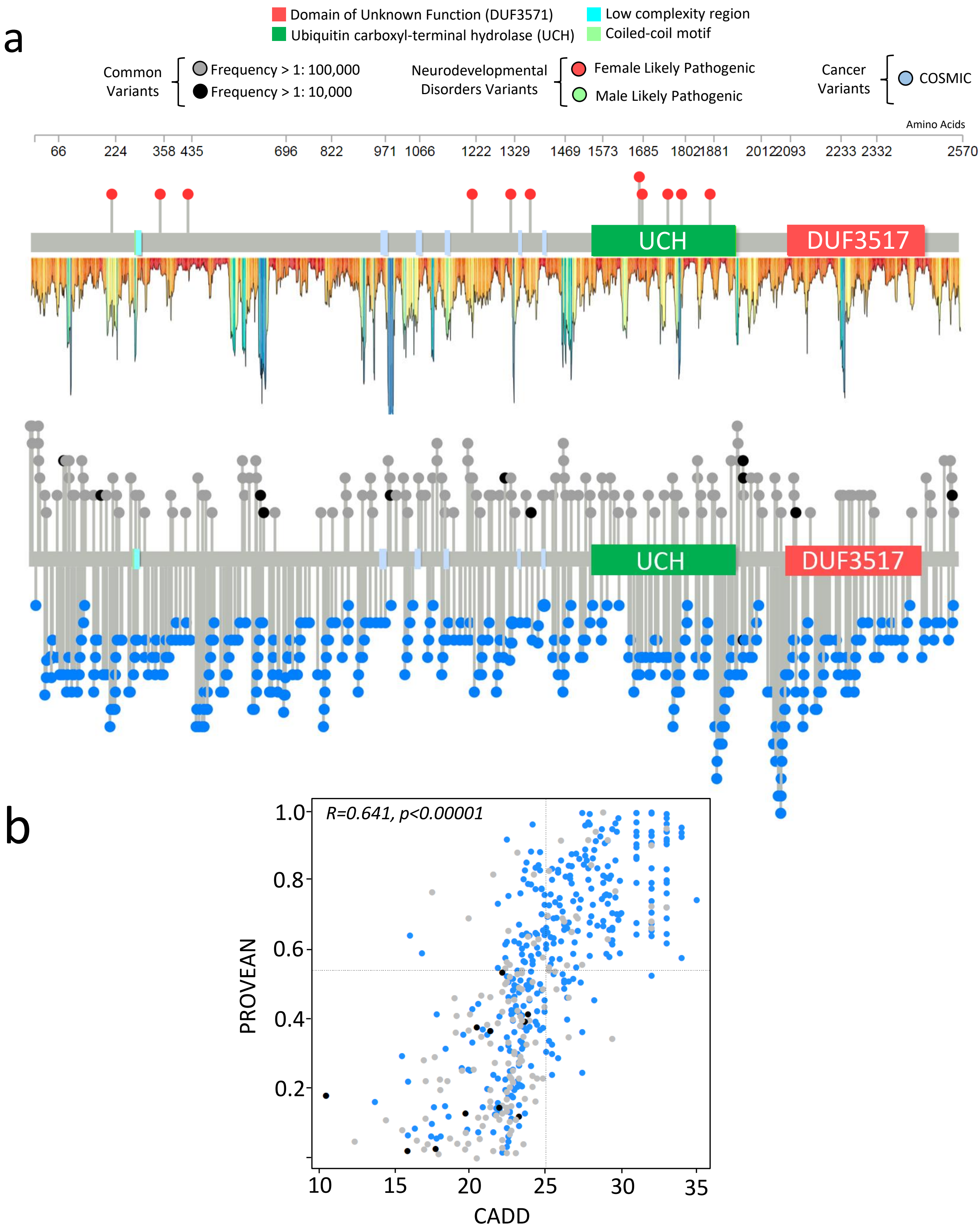


**Supplementary Figure 1. Distribution and classification of common and somatic cancer missense variants in USP9X.** (a) Location of female likely pathogenic variants on the USP9X protein structure. USP9X variation tolerance landscape is provided (see Materials and Methods). Locations of common missense variants extracted from GnomAD V2 data base (Frequency > 1: 100,000; n=159) and missense somatic cancer variants extracted from COSMIC data base (n=358) are shown for comparison. (b) Comparison of CADD and PROVEAN scores to assess pathogenicity (CADD >25, PROVEAN >0.565). Scores of common variants are significantly correlated (Pearson's correlation given). Around 45% of COSMIC variants are located in the pathogenic quadrant. Colour scheme as in (a).

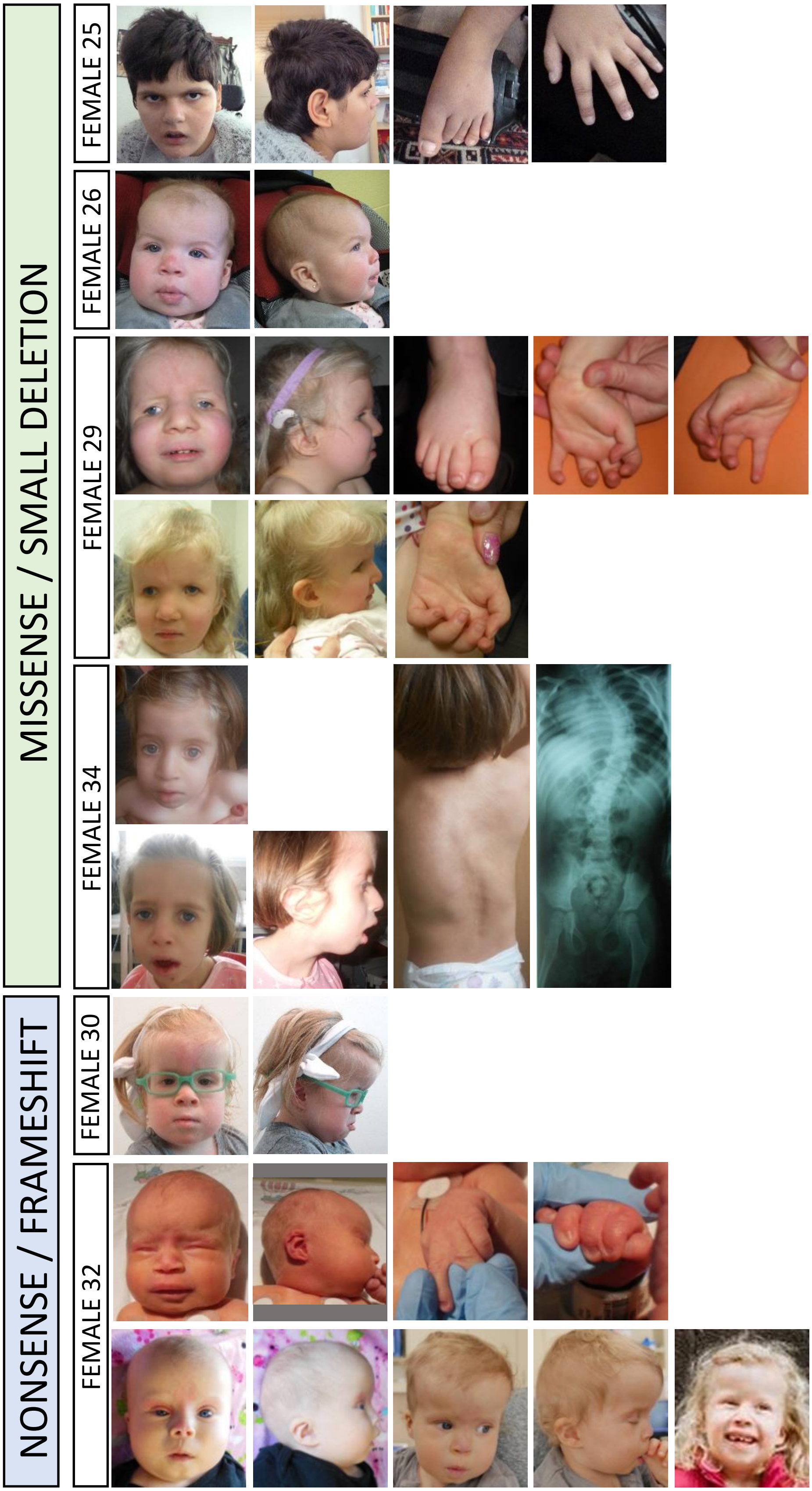


**Supplementary Figure 2. Facial and other dysmorphisms of female individuals with USP9X variants**. Note Female 30 carries a nonsense variant, Female 32 carries a frame shift variant, whilst all other individuals carry missense or singe amino acid deletion variants. Note common craniofacial features including deep-set eyes, telecanthus, blepharophimosis, a broad nasal tip with wide alae and short collumnella, and low set and dysplastic ears; overlapping toes in Females 25 and 29; digital defects in Females 25. 29 and 32 including tapered fingers, hypoplastic thumbs and thenar eminences among others; and scoliosis in Female 34.

a

| Case ID | Inheritance | Genomic | cDNA | Protien | Reference |
|---|---|---|---|---|---|
| Female 1 | De novo | g.41027389C>T | c.2554C>T | p.Arg852* | Reijnders et al 2015 |
| Female 2 | Maternal* | g.41047364T>A | c.3804T>A | p.Tyr1268* | Reijnders et al 2015 |
| Female 3 | De novo | g.41031089A>G | c.3028-2A>G | p.Val1009Ilefs*4 | Reijnders et al 2015 |
| Female 4 | De novo | g.41029255insA | c.2644_2645insA | p.Arg882Glnfs*3 | Reijnders et al 2015 |
| Female 5 | De novo | g.41082619-41090198del | n/a | n/a | Reijnders et al 2015 |
| Female 6 | De novo | g.40926769-41148506del | n/a | n/a | Reijnders et al 2015 |
| Female 7 | De novo | g.41025124G>T | c.1986-1G>T | p.Phe663Sfs*1 | Reijnders et al 2015 |
| Female 9 | De novo | g.41055608insGG | c.4082insGG | p.Ser1363Glyfs*18 | Reijnders et al 2015 |
| Female 10 | De novo | g.41089767-41089781del | c.7493delAAGATGCTCCAGATG | p.Asp2499_Glu2503del | Reijnders et al 2015 |
| Female 11 | De novo | g.41047323C>T | c.3763C>T | p.Gln1255* | Reijnders et al 2015 |
| Female 12 | De novo | g.41000634C>T | c.1111C>T | p.Arg371* | Reijnders et al 2015 |
| Female 13 | De novo | g.41000677delG | c.1154delG | p.Met386Trpfs*13 | Reijnders et al 2015 |
| Female 14 | De novo | g.41047269delT | c.3709delT | p.Cys1237Valfs*2 | Reijnders et al 2015 |
| Female 15 | De novo | g.40998091-41057271del | n/a | n/a | Reijnders et al 2015 |
| Female 16 | De novo | g.41055581dupT | c.4055dupT | p.Phe1353Leufs*18 | Reijnders et al 2015 |
| Female 17 | De novo | g.41032491-41059516del | n/a | n/a | Reijnders et al 2015 |
| Female 18 | De novo | g.41043878delG | c.3508delG | p.Val1170Trpfs*9 | Sinthuwiwat et al 2018 |
| Female 19 | De novo | g.41051522delACTCT | c.4068_4072del | p.Leu1357Tyrfs*12 | Tsurusaki et al 2019 |
| Female 20 | De novo | g.41002583C>T | c.1201C>T | p.Arg401* | Tsurusaki et al 2019 |
| Female 40 | De novo | g.41056674dupC | c.4291insC | Leu1431Profs*14 | Vianna et al 2020 |
| Female 30 | De novo | g.41000663G>A | c.1140G>A | p.Trp380* | This study |
| Female 31 | Maternal** | g.40996263C>T | c.642C>T | p.Arg215* | This study |
| Female 32 | De Novo | g.41007805dupA | c.1598_1599insA | p.Ile535Asnfs*11 | This study |

b

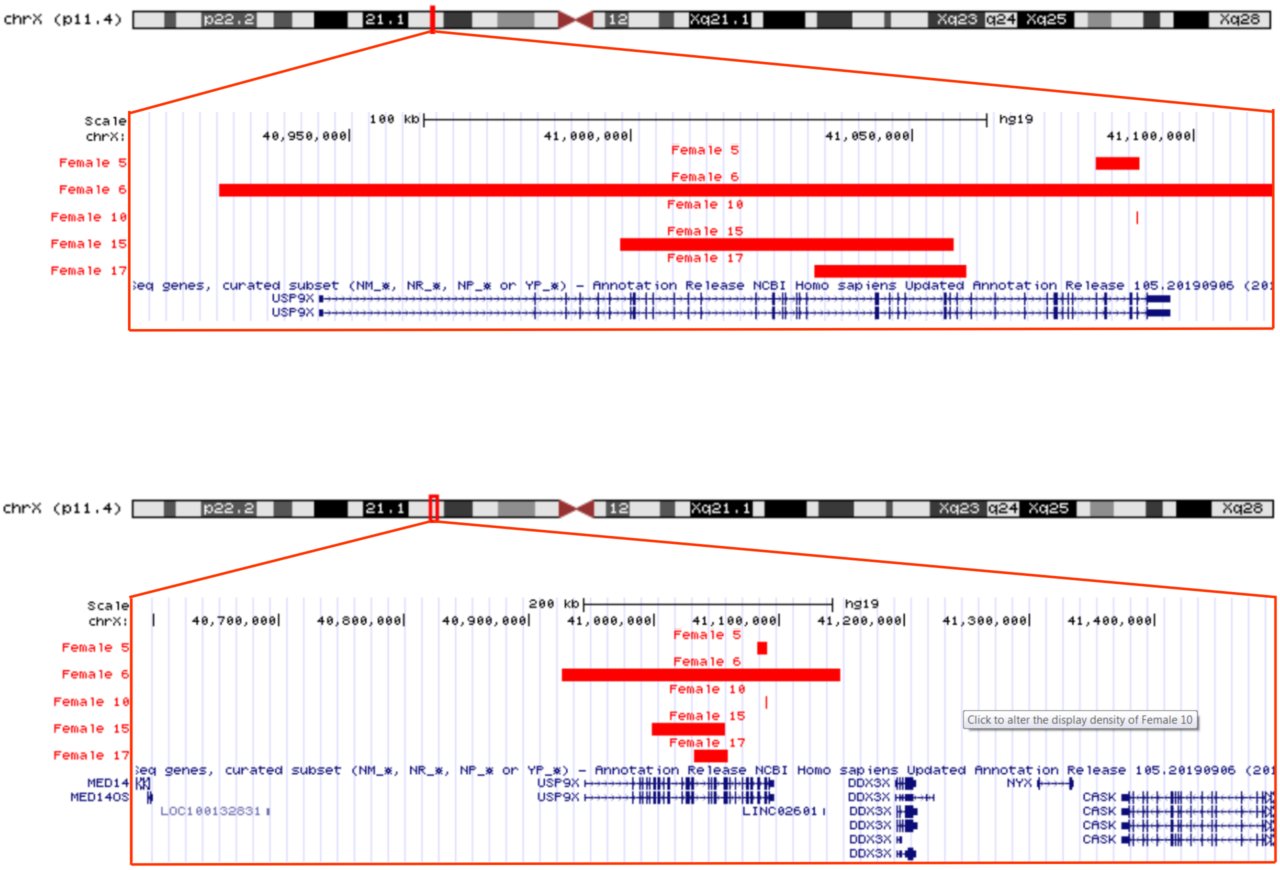


**Supplementary Figure 3. Bona fide complete loss of function USP9X alleles associated with female NDDs.** (a) Table of all known gene deletion, frameshift and nonsense variants associated with *USP9X* female NDDs. This includes 20 previously published and 3 novel variants reported in this study. * Inherited from a mosaic mother. **Mosaicism and X-inactivation status of mother unknown. (b) UCSC screen shots with coordinates of gene deletion variants tracked.
