# Supplementary material for: Missense variant contribution to USP9X-female syndrome
Source: NPJ Genom Med. 2020 Dec 9;5:53. doi: 10.1038/s41525-020-00162-9 (PMC7725775; doi:10.1038/s41525-020-00162-9)
Supplement: Supplementary file 3 — Reporting Summary [file 41525_2020_162_MOESM3_ESM.pdf]

nature research

Corresponding author(s): Jozef Gecz
Lachlan Jolly

Last updated by author(s): 2020/10/09

Reporting Summary

Nature Research wishes to improve the reproducibility of the work that we publish. This form provides structure for consistency and transparency in reporting. For further information on Nature Research policies, see our Editorial Policies and the Editorial Policy Checklist.

Statistics

For all statistical analyses, confirm that the following items are present in the figure legend, table legend, main text, or Methods section.

n/a	Confirmed	
☐	☒	The exact sample size (*n*) for each experimental group/condition, given as a discrete number and unit of measurement
☒	☐	A statement on whether measurements were taken from distinct samples or whether the same sample was measured repeatedly
☐	☒	The statistical test(s) used AND whether they are one- or two-sided<br>*Only common tests should be described solely by name; describe more complex techniques in the Methods section.*
☒	☐	A description of all covariates tested
☒	☐	A description of any assumptions or corrections, such as tests of normality and adjustment for multiple comparisons
☐	☒	A full description of the statistical parameters including central tendency (e.g. means) or other basic estimates (e.g. regression coefficient) AND variation (e.g. standard deviation) or associated estimates of uncertainty (e.g. confidence intervals)
☒	☐	For null hypothesis testing, the test statistic (e.g. *F*, *t*, *r*) with confidence intervals, effect sizes, degrees of freedom and *P* value noted<br>*Give P values as exact values whenever suitable.*
☒	☐	For Bayesian analysis, information on the choice of priors and Markov chain Monte Carlo settings
☒	☐	For hierarchical and complex designs, identification of the appropriate level for tests and full reporting of outcomes
☒	☐	Estimates of effect sizes (e.g. Cohen's *d*, Pearson's *r*), indicating how they were calculated

Our web collection on statistics for biologists contains articles on many of the points above.

Software and code

Policy information about availability of computer code

Data collection	We extracted common USP9X varinats from Genome Aggregation Database (gnomAD V2.1.1) and USP9X cancer variants from Catalogue of Somatic Mutations in Cancer (COSMIC V90) database. Structual modelling was perfromed using PyMol software (Schrödinger).
Data analysis	Two-proportion z-test for enrichment of variants in the catalytic domain was conducted using http://www.sthda.com/english/wiki/two-proportions-z-test-in-r. Variant predictions were performed using Annovar accessed via the webserver http://wannovar.wglab.org/. 2-tailed equal variance Student's t-test were conducted with microsoft excel. USP9X tolerance to variation landscape was outputted using the Metadome Version 1.0.1 webserver at https://stuart.radboudumc.nl/metadome/dashboard.

For manuscripts utilizing custom algorithms or software that are central to the research but not yet described in published literature, software must be made available to editors and reviewers. We strongly encourage code deposition in a community repository (e.g. GitHub). See the Nature Research guidelines for submitting code & software for further information.

Data

Policy information about availability of data

All manuscripts must include a data availability statement. This statement should provide the following information, where applicable:

- Accession codes, unique identifiers, or web links for publicly available datasets
- A list of figures that have associated raw data
- A description of any restrictions on data availability

Additional data and materials from this study are available from the authors on reasonable request, subject to compliance with our obligations under human research ethics.

Field-specific reporting

Please select the one below that is the best fit for your research. If you are not sure, read the appropriate sections before making your selection.

☒ Life sciences ☐ Behavioural & social sciences ☐ Ecological, evolutionary & environmental sciences

For a reference copy of the document with all sections, see nature.com/documents/nr-reporting-summary-flat.pdf

Life sciences study design

All studies must disclose on these points even when the disclosure is negative.

Sample size	Number of individual USP9X mutations analysed in this study was determined by availability of such data, i.e. the USP9X cohorts were assembled retrospectively. Number of comon and cancer variants was determined by availability of such data within the Gnomad and COSMIC data bases listed above.
Data exclusions	For comparisons of USP9X varaints against gnomAD common varinst we excluded gnomAD common varinats with allele freqeuncy > 1:100,000 as gnomAD may contain ultra rare varaints of borderline pathogenicity. For spatial/structual comparison of USP9X variants woth COSMIC variants we excluded COSMIC variants with CADD score <30 so as to focus on those with strong evidence of pathogenicity
Replication	Clinical phenotype of our USP9X female cohorts was compared to other USP9X female and male cohorts.
Randomization	Participants were grouped based on sex, and USP9X variant type.
Blinding	Blinding was not possible as numbers of participants and the variants in each cohort is are identifiable parameters.

Reporting for specific materials, systems and methods

We require information from authors about some types of materials, experimental systems and methods used in many studies. Here, indicate whether each material, system or method listed is relevant to your study. If you are not sure if a list item applies to your research, read the appropriate section before selecting a response.

Materials & experimental systems

n/a	Involved in the study	
☒	☐	Antibodies
☒	☐	Eukaryotic cell lines
☒	☐	Palaeontology and archaeology
☒	☐	Animals and other organisms
☐	☒	Human research participants
☒	☐	Clinical data
☒	☐	Dual use research of concern

Methods

n/a	Involved in the study	
☒	☐	ChIP-seq
☒	☐	Flow cytometry
☐	☒	MRI-based neuroimaging

Human research participants

Policy information about studies involving human research participants

Population characteristics	Female individuals with USP9X variants
Recruitment	Retrospectively recruited
Ethics oversight	Womens and Children's Health Network, Adelaide, Australia.

Note that full information on the approval of the study protocol must also be provided in the manuscript.

Magnetic resonance imaging

Experimental design

Design type	Resting
Design specifications	Varaiable. Data collected Retrospectively.
Behavioral performance measures	N/A

Acquisition

Imaging type(s): Varaiable. Data collected Retrospectively.

Field strength: Varaiable. Data collected Retrospectively.

Sequence & imaging parameters: Varaiable. Data collected Retrospectively.

Area of acquisition: Varaiable. Data collected Retrospectively.

Diffusion MRI: ☐ Used ☒ Not used

Preprocessing

Preprocessing software: Varaiable. Data collected Retrospectively.

Normalization: Varaiable. Data collected Retrospectively.

Normalization template: Varaiable. Data collected Retrospectively.

Noise and artifact removal: Varaiable. Data collected Retrospectively.

Volume censoring: Varaiable. Data collected Retrospectively.

Statistical modeling & inference

Model type and settings: N/A

Effect(s) tested: N/A

Specify type of analysis: ☒ Whole brain ☐ ROI-based ☐ Both

Statistic type for inference (See Eklund et al. 2016): N/A

Correction: N/A

Models & analysis

n/a	Involved in the study
☒	☐ Functional and/or effective connectivity
☒	☐ Graph analysis
☒	☐ Multivariate modeling or predictive analysis
